# Cross-interaction of tau PET tracers with monoamine oxidase B: evidence from in silico modelling and in vivo imaging

**DOI:** 10.1007/s00259-019-04305-8

**Published:** 2019-03-27

**Authors:** N. Arul Murugan, Konstantinos Chiotis, Elena Rodriguez-Vieitez, Laetitia Lemoine, Hans Ågren, Agneta Nordberg

**Affiliations:** 1 Department of Theoretical Chemistry and Biology, School of Engineering Sciences in Chemistry, Biotechnology and Health, KTH Royal Institute of Technology, AlbaNova University Center, S-106 91 Stockholm, Sweden; 2 Department of Neurobiology, Care Sciences and Society, Center for Alzheimer Research, Division of Clinical Geriatrics, Karolinska Institutet, Stockholm, Sweden; 3 Theme Neurology, Karolinska University Hospital, Stockholm, Sweden; 4 Department of Physics and Astronomy, Uppsala University, Box 516, SE-751 20 Uppsala, Sweden; 5 Theme Aging, Karolinska University Hospital, Stockholm, Sweden

**Keywords:** Tau PET imaging, Off-target binding, Monoamine oxidase B, Alzheimer’s disease, Molecular docking, Binding free energy calculations

## Abstract

**Purpose:**

Several tracers have been designed for tracking the abnormal accumulation of tau pathology in vivo. Recently, concerns have been raised about the sources of off-target binding for these tracers; inconclusive data propose binding for some tracers to monoamine oxidase B (MAO-B).

**Methods:**

Molecular docking and dynamics simulations were used to estimate the affinity and free energy for the binding of several tau tracers (FDDNP, THK523, THK5105, THK5317, THK5351, T807 [aka AV-1451, flortaucipir], T808, PBB3, RO-948, MK-6240, JNJ-311 and PI-2620) to MAO-B. These values were then compared with those for safinamide (MAO-B inhibitor). PET imaging was used with the tau tracer [^18^F]THK5317 and the MAO-B tracer [^11^C]DED in five patients with Alzheimer’s disease to investigate the MAO-B binding component of this first generation tau tracer in vivo.

**Results:**

The computational modelling studies identified a binding site for all the tau tracers on MAO-B; this was the same site as that for safinamide. The binding affinity and free energy of binding for the tau tracers to MAO-B was substantial and in a similar range to those for safinamide. The most recently developed tau tracers MK-6240, JNJ-311 and PI-2620 appeared, in silico, to have the lowest relative affinity for MAO-B. The in vivo investigations found that the regional distribution of binding for [^18^F]THK5317 was different from that for [^11^C]DED, although areas of suspected off-target [^18^F]THK5317 binding were detected. The binding relationship between [^18^F]THK5317 and [^11^C]DED depended on the availability of the MAO-B enzyme.

**Conclusions:**

The developed tau tracers show in silico and in vivo evidence of cross-interaction with MAO-B; the MAO-B component of the tracer binding was dependent on the regional concentration of the enzyme.

**Electronic supplementary material:**

The online version of this article (10.1007/s00259-019-04305-8) contains supplementary material, which is available to authorized users.

## Introduction

Alzheimer’s disease (AD) is characterized by the accumulation of insoluble fibril aggregates of amyloid-beta and tau proteins in the brains of patients. The development of tau-specific PET tracers is now gaining in interest since post-mortem studies have indicated that tau pathology seems to track cognitive deterioration better than amyloid-beta deposition, and has been observed in both AD and non-AD-related neurodegenerative diseases (i.e. primary tauopathies) [1]. However, tracers for tau pathology are only just emerging and thorough investigation of their binding mechanisms, using ante-/post-mortem data, has not yet been carried out, especially with regard to off-target binding.

The tracers THK5317, THK5351, T807 (aka AV-1451, flortaucipir) and PBB3 are to date the most widely studied tau tracers. In vitro, these tracers have shown high affinity and selectivity for tau deposits [2–5]. When injected in vivo into patients with AD or non-AD tauopathies, they have shown extensive binding in the relevant brain areas and clear discrimination from groups of cognitively normal volunteers [4, 6–9]. However, all these tracers also showed substantial binding in areas not primarily related to the accumulation of tau pathology in AD (e.g. the basal ganglia) [6, 10, 11]. For tracers of the THK family and T807, the signal in the basal ganglia has been preliminarily attributed to binding to monoamine oxidase B (MAO-B) [12–14]. However, a recent in vitro study has suggested that the affinity of the tracers for the MAO-B enzyme is relatively low (i.e. low Ki for [^3^H]deuterium-L-deprenyl (DED)), which would theoretically not allow PET to detect this binding [3]. After the THK family, T807 and PBB3 tracers, second generation tau tracers that are thought to have less extensive off-target binding started to emerge (i.e. RO-948 [RO69558948], GTP-1, MK-6240, JNJ-311 [JNJ64349311], PI-2620); however, little in vivo data have been published for these so far [15, 16]. Overall, the exact contribution of MAO-B binding to the total off-target signal, and the brain areas that are particularly vulnerable to this off-target signal, remain to be determined for the available tau tracers.

The aim of this study was twofold. The first part aimed, with the use of computational modelling, to investigate the potential cross-interaction of the developed tau-specific tracers with MAO-B; the binding affinity of the tau tracers to MAO-B was determined and compared with that of an MAO-B inhibitor using in silico simulations of the underlying molecular interactions. The second part aimed to assess the translation of the in silico findings in an in vivo paradigm. We evaluated the MAO-B binding component of a tau tracer in vivo, using a multimodal PET design in which the same patients were scanned sequentially with a MAO-B tracer ([^11^C]DED) and a tau tracer ([^18^F]THK5317).

## Materials and methods

### Computational modelling of the cross-interaction between the tracers and MAO-B

Computational modelling was employed to calculate the relative binding affinity of the tau tracers to the MAO-B target. Molecular docking was employed to identify the most stable binding modes and poses for various ligands. The molecular dynamics approach was used to study the stability of the complexes under ambient conditions, and the molecular mechanics-generalized Born surface area (MM-GBSA) approach was applied to calculate the free energy of binding to MAO-B for these small molecules. For the modelling studies, we employed the chemical structures of the tau tracers FDDNP (a tracer with affinity for both amyloid-beta and tau), THK523, THK5105, THK5317, THK5351, T807, T808, PBB3, RO-948, MK-6240, JNJ-311 and PI-2620 [17], and the reversible MAO-B inhibitor safinamide [18].

It should be noted that the T808 structure was selected instead of the structure of GTP-1 (which has the same chemical structure to the Τ808, with the exception of two hydrogen atoms that were replaced by deuterium), since the two structures are treated by the force-field methods essentially in the same way; the Lennard-Jones parameters and atomic charge for deuterium are the same as that for hydrogen.

#### Molecular docking

The structures of all the ligands mentioned above (tau tracers and safinamide) were built using Molden software. The geometry was optimized by the B3LYP/6–31+G* level of theory in the gaussian09 software [19]. The optimized molecular structures were used in the docking simulation with the MAO-B target, the structure of which was obtained from a protein database (PDB reference ID 2V5Z) [20]. In this crystal structure, MAO-B was co-crystallized with safinamide. MAO-B exists in a dimeric form and only chain A was used for the docking studies; as the binding site is not located in the interfacial region, a monomer model was considered sufficient. Autodock4.0 [21] was used to carry out the molecular docking simulations. The size of the grid box was x = 63, y = 75, z = 79 Å. The number of grid points was 170x230x210, since the default grid spacing (which is 0.375 Å) was used. This was to make sure that it included the binding site reported previously along with any other potential surface binding sites. The docking simulation also included the cofactor flavin adenine dinucleotide (FAD) in the binding site. A total of 500 low energy configurations were determined for the molecules in the MAO-B binding site. The configuration corresponding to the lowest binding energy for the complex was used as the input for subsequent molecular dynamics simulations. The binding energies of the most stable complex structures were used for analysis of inhibition constants. In particular, blind docking was employed for identifying potential binding sites for these ligands, other than the substrate binding site discussed in the literature [22], within the MAO-B target.

#### Molecular dynamics and free energy calculations

The molecular dynamics simulations were carried out using the Amber/14 software [23]. The charges for the ligands were obtained from the B3LYP/6–31+G* level of theory and the CHELPG method as implemented in gaussian09 [19]. The ligands were described using the general amber force field. The charges and force-field libraries for the FAD cofactor were obtained in the same way; its position in the protein was based on the crystal structure. The protein was described using the FF99SB force field, and the TIP3P model was used to describe the water solvent. All MAO-B:ligand complexes were solvated with around 25,800 solvent molecules. Initially, minimisation runs were carried out for all the MAO-B:ligand complexes, and then heating runs were performed to bring the systems to room temperature and 1 atm pressure. We have used the MAO-B:ligand structure as obtained from the minimisation run for computing the protein-ligand interaction diagram. The temperature and pressure were controlled by connecting the system to the Langevin thermostat (collision frequency 5 ps^−1^) and Berendsens barostat, respectively. The time step for the integration of equation of motion was set to 2 fs and the time scale for the equilibration runs was 5 ns. The convergence of properties such as density and energy was analysed to make sure the systems reached the equilibrium state. The time scale for the production runs was 30 ns. The 1000 configurations from the last 5 ns of molecular dynamics simulations were used for the binding free energy calculations. We used the molecular dynamics simulations to investigate the stability of the protein:ligand complexes. In general, unstable complexes dissociate during the course of the simulations and in the current study all the tracers: MAO-B complexes were found to be stable. The stability of the MAO-B:ligand complexes was assessed by computing the root mean square displacement (RMSD) for the ligands.

While molecular docking results reproduce the binding pose and mode of the ligands in the enzyme binding sites, the binding affinities computed from molecular docking are based on the most stable complex structure, which does not account for the temperature or sampling effects. Moreover, the conformational flexibility of the protein is not accounted for in this approach. Thus, in order to predict the relative binding affinity of the ligands more accurately, the free energies of binding were computed, using the MM-GBSA approach [24], for various configurations from the molecular dynamics trajectories. In this approach, the free energy for the association of the ligands with enzymes in solvents is computed, and the solvents are described implicitly. The protein:ligand electrostatic energies in solvents were computed by solving the generalized Born equation. The non-polar contributions to solvation free energies were computed using the solvent accessible surface area (SASA). Overall, the computed free energy of binding includes van der Waals, electrostatic and polar and non-polar solvation free energies along with entropic contributions. As the entropy calculations are both memory intensive and computationally demanding, these calculations were carried out for only 50 configurations. The python post-processing script MMGBSA.py [25] was used to calculate all these contributions to the total binding free energy. In addition, the residue-wise contributions to the total free energy was calculated for most of the ligands (i.e. safinamide, THK5317, THK5351, PBB3, T807, RO-948, MK-6240, JNJ-311 and PI-2620) to investigate how much the co-factor FAD contributed to the total binding free energy and thus to the overall stability of the complexes. Because the binding free energies are quantitatively larger than the free energies from molecular docking and the absolute values are not of much significance, we only analysed the relative binding free energy of the ligands.

### MAO-B component of tracer binding in vivo

We retrospectively compared in vivo tau [^18^F]THK5317 and [^11^C]DED (i.e. the tracer analogue of the irreversible MAO-B inhibitor selegiline) PET images from a group of five AD patients, each of whom had undergone both [^18^F]THK5317 and [^11^C]DED scans on separate occasions, with the aim of investigating the extent to which the in vivo [^18^F]THK5317 binding was due to binding to MAO-B. Voxel-wise comparisons between [^18^F]THK5317 and [^11^C]DED were carried out, between and within each patient. Analyses were carried out to investigate whether the strength of the association between [^18^F]THK5317 and [^11^C]DED differed between regions of high (sub-cortical regions including the basal ganglia and thalami) and low (isocortex) MAO-B levels, based on previous reports on MAO-B brain concentrations in post-mortem investigations [26].

#### Participants

Each of the five patients with AD (aged 55–74 years) had previously undergone MRI, and [^11^C]DED [27], [^11^C]PIB and, subsequently, as part of a separate project, [^18^F]THK5317 PET imaging. Because the [^11^C]DED and the [^18^F]THK5317 PET imaging were performed for separate projects, the interval between PET scans ranged from 0.8 to 2.3 years, and the data were studied retrospectively. All patients had been initially referred to the Cognitive Clinic at the Theme Aging, Karolinska University Hospital, Stockholm, Sweden, where they underwent thorough clinical investigation, as previously described [27]. Two of the patients had a clinical diagnosis of probable AD [28] and three of mild cognitive impairment [29]. According to the new research diagnostic criteria [30], and based on the positivity of all patients in their amyloid-beta PET scans ([^11^C]PIB), the patients were re-classified as having AD dementia (*n* = 2) and prodromal AD (*n* = 3), respectively. One patient with a clinical diagnosis of prodromal AD at the time of [^11^C]DED PET was rediagnosed as AD dementia at the time of [^18^F]THK5317 PET investigation (patient 3). Information with regard to the clinical diagnosis, global cognitive performance (mini mental state examination (MMSE) score) and treatment of all participants at the time points of [^11^C]DED and [^18^F]THK5317 PET investigations is presented in Supplementary Table 3.

#### PET and MRI image acquisition and processing

Participants underwent 60-min dynamic [^11^C]DED and [^18^F]THK5317 PET scans at the Uppsala PET Centre, Uppsala University (Sweden), following previously reported procedures for radiotracer administration, PET image acquisition, reconstruction and motion correction [6, 27, 31]. The [^11^C]DED scans were performed on GE discovery ST PET/CT (patients 1, 3 and 4) and ECAT EXACT HR+ (Siemens/CTI) (patients 2 and 5) scanners. All [^18^F]THK5317 PET scans were performed on ECAT EXACT HR+ (Siemens/CTI) scanners. The [^11^C]DED data on the ECAT EXACT HR+ system was reconstructed with filtered back projection (FBP), Hann filter with 4-mm full width at half maximum (FWHM) and zoom 2.5, while the [^11^C]DED data on the Discovery ST PET/CT system was reconstructed with 3D brain Fourier rebinning FBP, enhanced Hann filter with 6.4 mm FWHM. All [^18^F]THK5317 data on the ECAT EXACT HR+ system was reconstructed with ordered-subsets expectation-maximisation, 6/8 Hann filter with 4 mm FWHM and zoom 2.5. The differences in reconstruction methods for the ECAT EXACT HR+ system were due to the different scanner software at the two time points. By employing a NEMA image quality phantom, we selected reconstruction parameters methods of the GE discovery ST PET/CT, which matched best the reconstruction that was already applied to the ECAT EXACT HR+ data, for enabling the comparability of the resulting images (unpublished work). For each participant, a structural 3D T1 magnetization-prepared rapid-acquisition gradient-echo sequence MRI image was also acquired.

The individual dynamic [^18^F]THK5317 images were co-registered onto the individual T1-weighted images and the distribution volume ratio (DVR) [^18^F]THK5317 images were created based on the reference Logan graphical method over the 30–60 min scan interval, with cerebellar grey matter (GM) used as a reference, as previously described [6] (PMOD v. 3.5 Technologies Ltd., Adliswil, Switzerland). For [^11^C]DED PET quantification, a modified reference Patlak model was applied to the 20–60 min dynamic [^11^C]DED PET images using the cerebellar GM as the “modified” reference region, as previously reported [27, 31], to generate individual parametric Patlak slope images (units: min^−1^). Although the parametric [^11^C]DED images were originally generated in the native PET space, the images were projected onto the individual T1-weighted MRI images, with an additional co-registration step (SPM8), in order to directly compare [^11^C]DED binding with [^18^F]THK5317 binding. Prior to performing voxel-wise analyses, the co-registered [^11^C]DED and [^18^F]THK5317 images were smoothed (FWHM = 4 mm in all directions) and rescaled, in order to reduce the total amount of voxels per image, to a final 4-mm voxel size.

#### Regions of interest

Each individual T1-weighted MRI image was divided into GM and white matter tissue classes using the SPM8 software unified segmentation, and a binary GM mask was created from the resultant probabilistic GM map (threshold = 0.5). The inverse nonlinear transformation from this segmentation step was used to warp the simplified probabilistic Hammers atlas into each individual’s native T1 space. The resulting individual atlases were then multiplied using the corresponding binarised probabilistic GM mask, to obtain individual GM atlases. The individual atlases were used to sample every GM voxel of the parametric [^18^F]THK5317 DVR and [^11^C]DED slope images. The voxels were classified to an isocortical region of interest (ROI) (voxels mapping the isocortical areas of the temporal, frontal, parietal and occipital lobes; low MAO-B ROIs) and a subcortical ROI (voxels mapping the basal ganglia and thalami; high MAO-B ROIs).

#### Statistical analysis

Voxel-wise correlations between [^11^C]DED and [^18^F]THK5317 were carried out using Spearman correlation analysis within patients for the two ROIs. In addition, a linear mixed-effects model was used to analyse the effect of [^11^C]DED binding on [^18^F]THK5317 binding while incorporating the influence of ROIs and the patient’s average [^11^C]DED binding, as follows:

$$ THK 5317= DED+ ROI+{Patients}^{'}\  average\  DED+ DED: ROI\ (interaction)+ DED:{Patients}^{'}\  average\  DED\ (interaction)+ Random\ intercept\ \left( Patient\  ID: ROI\right)+\varepsilon $$

[^18^F]THK5317 binding was treated as the dependent variable, [^11^C]DED binding was a fixed-effects continuous variable, ROI was a fixed-effects nominal variable (isocortical vs sub-cortical), and each patient’s average GM [^11^C]DED binding was a fixed-effects continuous variable. A random intercept was incorporated for patient identification, nested for the two ROIs. For the linear mixed-effects model analysis, the threshold for statistical significance was set at *p* < 0.05. All statistical analyses were carried out with R v.3.1.3 software. Graphical representations were made with the ggplot2 package v.1.0.1, as implemented in R v.3.1.3 software.

## Results

### Computational modelling of the cross-interaction between the tracers and MAO-B

#### Molecular docking

**Fig. 1:**
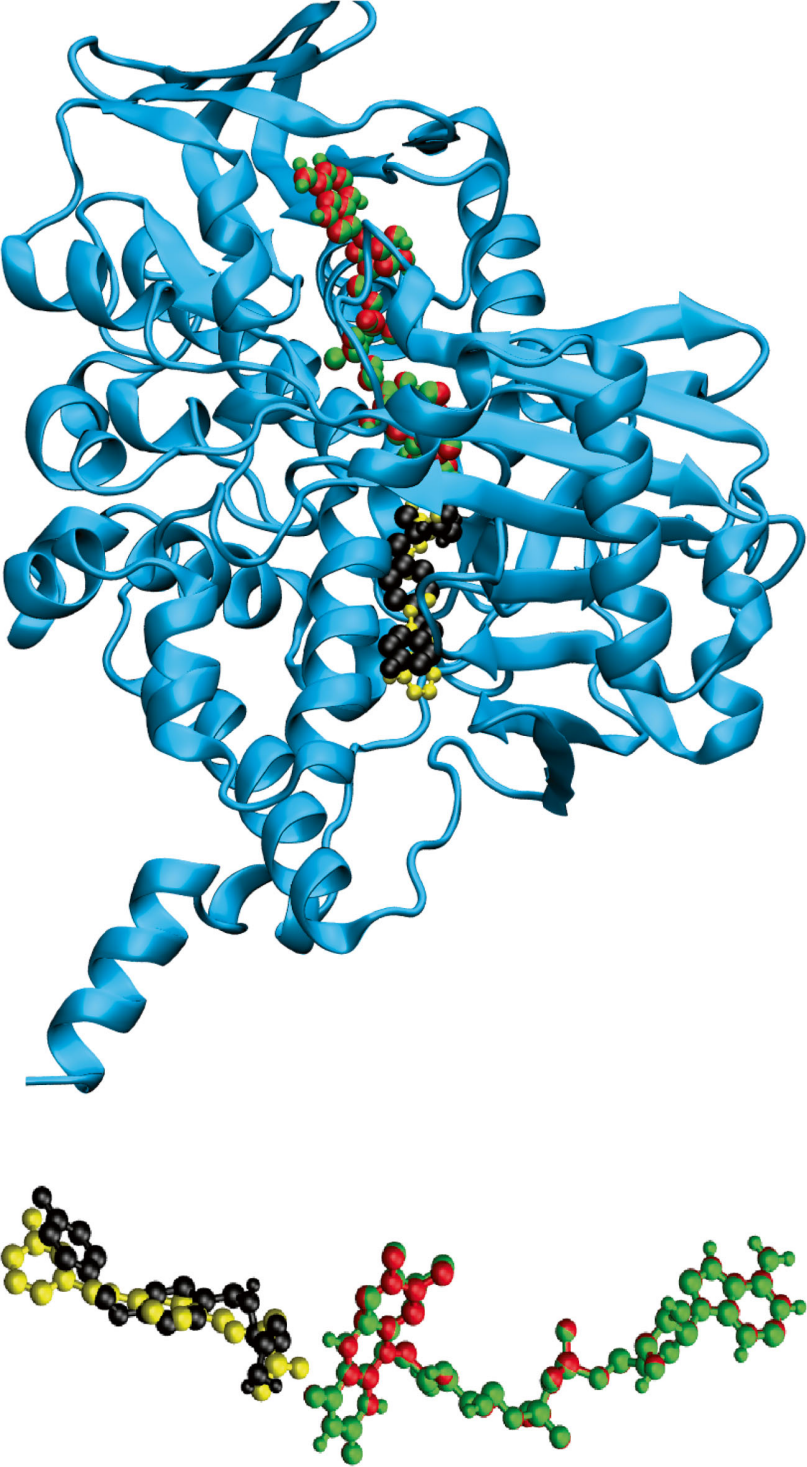
Structure of safinamide (crystal structure in *black*, docked structure in *yellow*) and of the FAD cofactor (crystal structure in *red*, docked structure in *green*), embedded into MAO-B (shown as a ribbon model in *cyan*). FAD = flavin adenine dinucleotide; MAO-B = monoamine oxidase B

In order to evaluate the ability of the molecular docking software to predict the binding site reliably, we superimposed the crystal structure of MAO-B:safinamide (as in 2V5Z) with the complex structure obtained from docking; the results are shown in Fig. 1. A reasonable overlap between the crystal and docked structures was observed. The FAD cofactor and the structure of MAO-B are also shown in Fig. 1.

Table 1 summarizes the estimates from the Autodock molecular docking tool for the binding affinity and inhibition constants for the MAO-B inhibitor safinamide and the tau tracers to the MAO-B target. Only results for the most stable MAO-B:ligand complexes are presented in Table 1. The binding affinity to MAO-B for all the tau tracers (−8.35 to −10.09 kcal/mol) was similar to that for safinamide (−9.64 kcal/mol). Further, the inhibition constants were in the nM range for all tau tracers.

**Table 1:** Binding affinities and inhibition constants for the monoamine oxidase-B (MAO-B) inhibitor safinamide and the studied tau PET tracers, calculated using molecular docking methods

Measure	Binding affinity (kcal/mol)	Inhibition constant, Ki
MAO-B inhibitor		
Safinamide	−9.64	86.21 nM
Tau tracers		
FDDNP	−9.56	98.77 nM
PBB3	−9.85	59.99 nM
T807	−9.50	108.17 nM
T808	−9.66	82.4 nM
THK5105	−10.09	40.37 nM
THK523	−9.17	190.90 nM
THK5317	−9.70	77.31 nM
THK5351	−9.54	102.46 nM
RO-948	−9.24	169.30 nM
MK-6240	−9.56	98.68 nM
JNJ-311	−8.35	758.04 nM
PI-2160	−9.23	172.96 nM

**Fig. 2:**
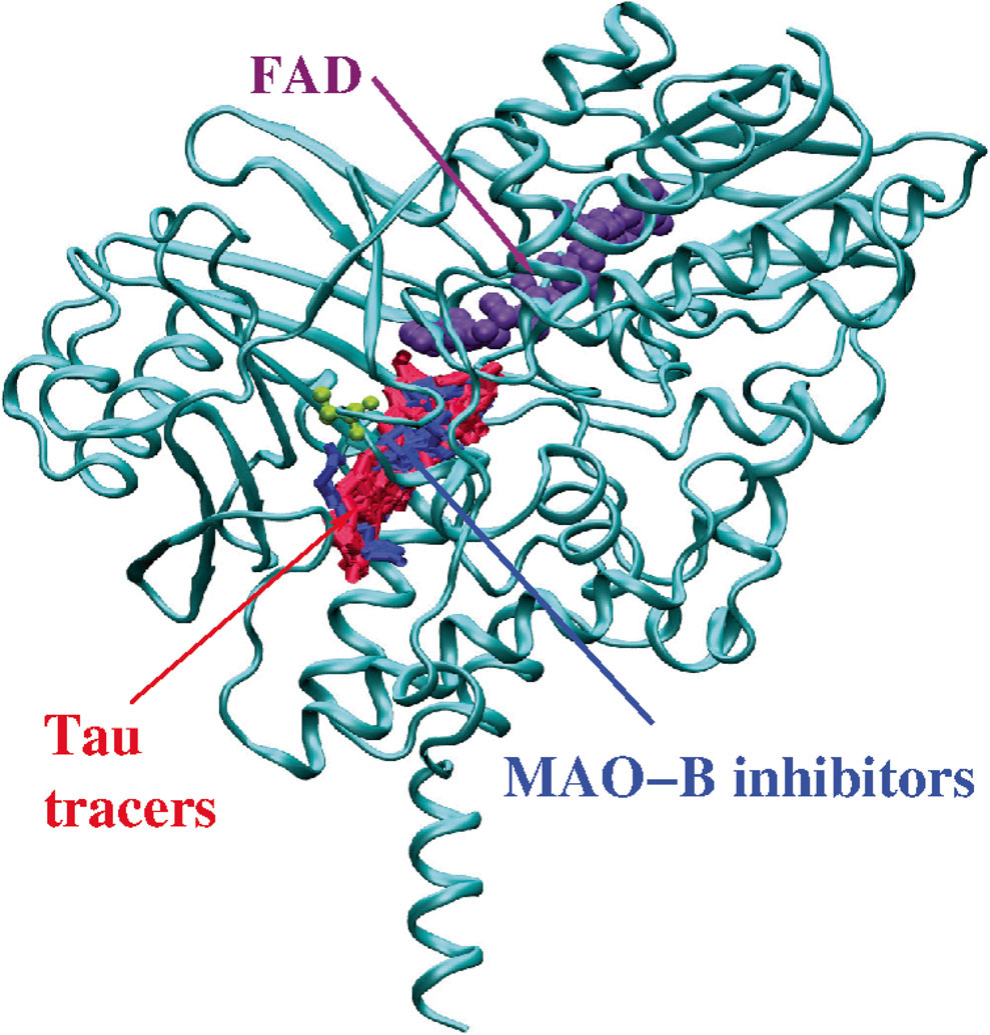
Structure of MAO-B (*light blue*) and its binding site for the MAO-B inhibitor safinamide (*dark blue*) and for the tau PET tracers (*red*); the FAD cofactor is shown in *purple*. The figure shows that the MAO-B inhibitors and tau tracers share the same binding site within the MAO-B molecule. FAD = flavin adenine dinucleotide; MAO-B = monoamine oxidase B

Because it was considered relevant to investigate whether these tau tracers also bound to the same site in MAO-B as the MAO-B inhibitor, we merged the binding pose for each of the tracers with that for safinamide and, as shown in Fig. 2, all compounds shared the same binding site. All the studied molecules bound to the substrate cavity site, and also partly occupied the entrance cavity site [22].

#### Molecular dynamics and free energy calculations

**Fig. 3:**
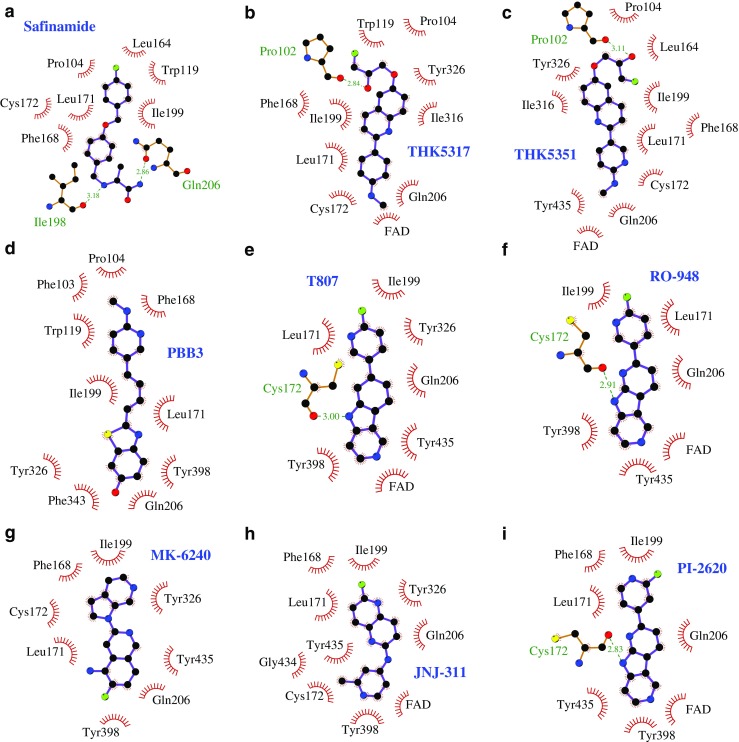
Protein:ligand interaction diagrams for **a** safinamide, **b** THK5317, **c** THK5351, **d** PBB3, **e** T807, **f** RO-948, **g** MK-6240, **h** JNJ-311 and **i** PI-2620. There is a hydrogen bond interaction between safinamide and the residues ILE198 and GLN206, in addition to hydrophobic interactions with various residues. There are hydrogen bond interactions between the tau tracers and specific residues, and some hydrophobic interactions with the residues; the interactions with the FAD cofactor are only hydrophobic. FAD = flavin adenine dinucleotide; MAO-B = monoamine oxidase B

Table 2 presents the binding free energies for various tau tracers and MAO-B inhibitor with the MAO-B target, computed using the MM-GBSA approach. The binding free energy of the reversible MAO-B inhibitor safinamide was −23.5 kcal/mol, which explains the high binding affinity of this compound to the MAO-B target. The protein:ligand interaction diagram for MAO-B:safinamide is shown in Fig. 3a. As can be appreciated, in addition to hydrophobic interactions between the safinamide and protein residues, there is a hydrogen-bonding interaction with two of the residues ILE198 and GLN206.

**Table 2:** Binding free energy (∆G_binding_) values for the monoamine oxidase B (MAO-B) inhibitor safinamide and the studied tau PET tracers binding with the MAO-B target

Measure	∆E_vdw_	∆E_elec_	∆G_GB_	∆G_SA_	-T∆S	∆G_binding_
MAO-B inhibitor						
Safinamide	−47.93	−20.11	29.79	−6.3	21.04	**−**23.51
Tau tracers						
FDDNP	−44.22	−7.48	21.79	−5.51	16.37	**−**19.05
PBB3	−46.61	−10.93	22.36	−6.00	18.13	**−**23.05
T807	−47.00	−13.56	23.98	−4.58	15.56	**−**25.60
T808	−49.10	−12.61	27.41	−5.73	20.79	**−**19.24
THK5105	−51.79	−15.40	30.21	−6.24	23.20	**−**20.02
THK523	−44.58	−13.07	25.22	−5.52	19.32	**−**18.63
THK5317	−48.54	−9.74	20.72	−6.19	20.87	**−**22.88
THK5351	−51.79	−15.40	30.21	−6.24	23.20	**−**20.02
RO-948	−46.71	−16.02	27.16	−4.52	20.42	**−**19.67
MK-6240	−43.27	−9.77	22.78	−4.87	18.95	**−**16.18
JNJ-311	−41.85	−6.55	24.93	−4.75	17.68	**−**10.54
PI-2620	−36.86	−9.24	25.63	−4.48	17.65	**−**7.30

The molecular mechanics-generalized Born surface area free energy calculations were carried out for configurations obtained using molecular dynamics. The binding free energy was computed using the equation: ∆G_binding_ = ∆E_vdw_ + ∆E_elec_ + ∆G_GB_ + ∆G_SA_ -T∆S, where ∆E_vdW_, ∆E_elec_, ∆G_GB_ and G_SA_ are van der Waals, electrostatic, polar and non-polar desolvation free energy terms and T∆S is the entropy (sum of translational, rotational and vibrational) contribution. All terms are in kcal/mol. The maximum standard error for the van der Waals, electrostatic, polar and non-polar free energy was 0.4 kcal/mol, while that for entropy was 0.7 kcal/mol

**Fig. 4:**
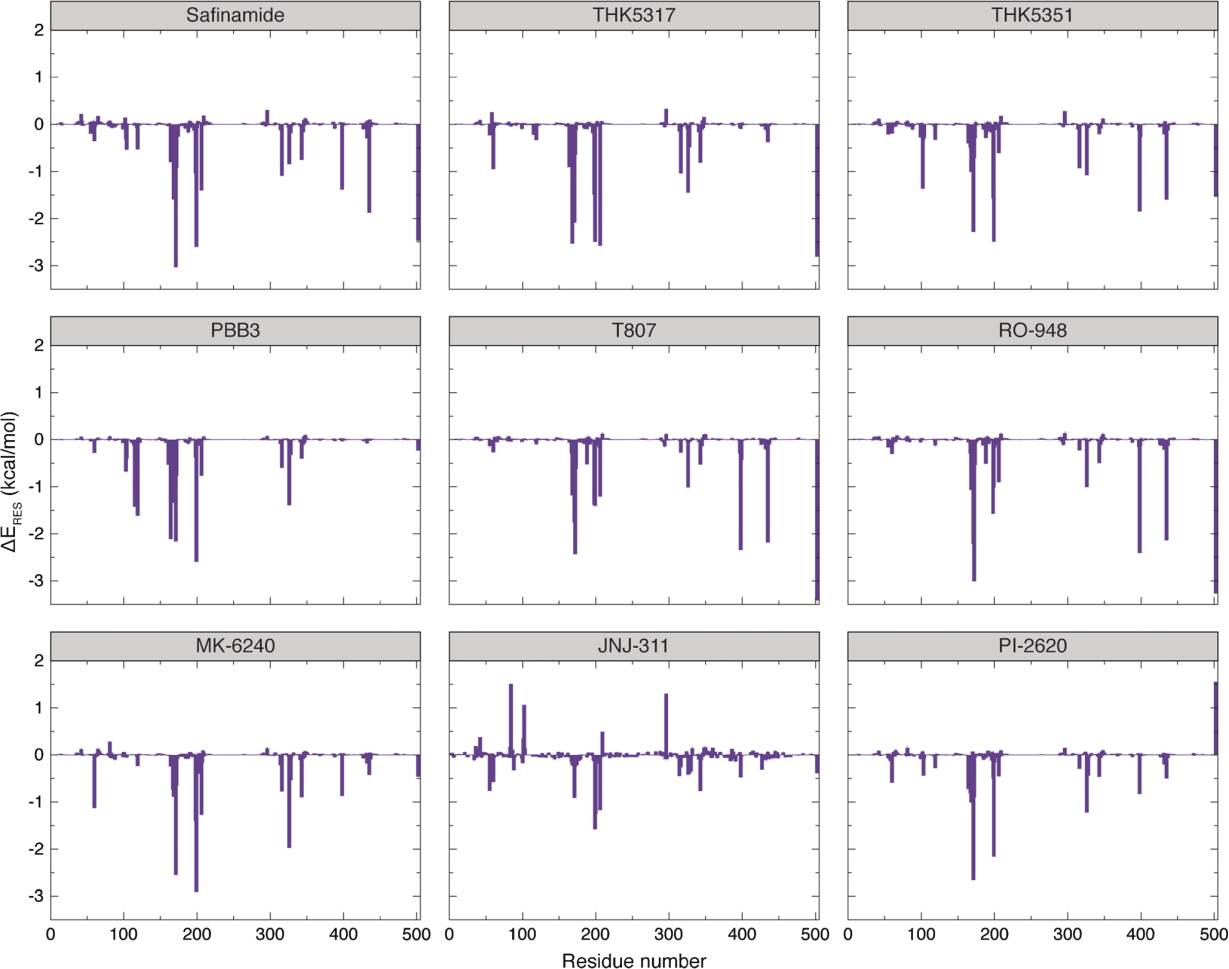
Residue-wise decomposition of free energy for the MAO-B inhibitor safinamide, and the tau tracers THK5317, THK5351, PBB3, T807, RO-948, MK-6240, JNJ-311 and PI-2620. The FAD cofactor contributes favourably to the complex formation with safinamide, and contributes significantly to the binding free energy for THK5317, THK5351 and T807. FAD = flavin adenine dinucleotide; MAO-B = monoamine oxidase B

The binding free energy values for the tau tracers (range − 10.54 to −25.60 kcal/mol) were comparable with that for the MAO-B inhibitor safinamide (−23.51 kcal/mol); MK-6240, JNJ-311 and PI-2620 had the lowest and T807 had the highest (in terms of magnitude) values for binding to MAO-B; THK523, T808 and RO-948 had free energy values intermediate between those of the first and second generation tracers (Table 2). In order to quantify the free energy contributions from various residues and the FAD cofactor, a decomposition analysis was performed for selected ligands. Figure 3 shows the MAO-B:ligand interaction diagrams for the association process of the MAO-B inhibitor safinamide and the tau tracers THK5317, THK5351, PBB3, T807, RO-948, MK-6240, JNJ-311 and PI-2620 with the MAO-B target, and Fig. 4 shows the residue-wise interactions contributing to the total free energy of binding. The similarities in the list of residues are noteworthy. The co-factor contributed greatly to the total binding free energy for the ligands safinamide, THK5317, THK5351 and T807 (as much as −2.0 to −3.5 kcal/mol). Although PBB3 occupies the same substrate-binding site as THK5351 and safinamide, the contribution from FAD was negligible for this ligand, with the residues HIE115 (−1.5 kcal/mol), PHE118 (−1.3 kcal/mol), TRP119 (−1.6 kcal/mol), ILE199 (−2.2 kcal/mol), LEU171 (−2.3 kcal/mol) and CYS172 (−0.8 kcal/mol) contributing dominantly in this case. As can be seen not all the residues seen in the protein-ligand interaction diagram are contributing dominantly in the residue-wise decomposition analysis. We recall that the protein:ligand interaction diagram was based on the minimum energy structure while here the residue-wise contributions are obtained as an average over many configurations from molecular dynamics trajectories. The main contributions to the interaction energy came from van der Waals’s interactions. It is worth recalling that, even in the case of tau fibrils, the hydrophobic interactions with beta-sheets are the driving force for the association process between the tracers and the fibrils.

### MAO-B component of tracer binding in vivo

**Fig. 5:**
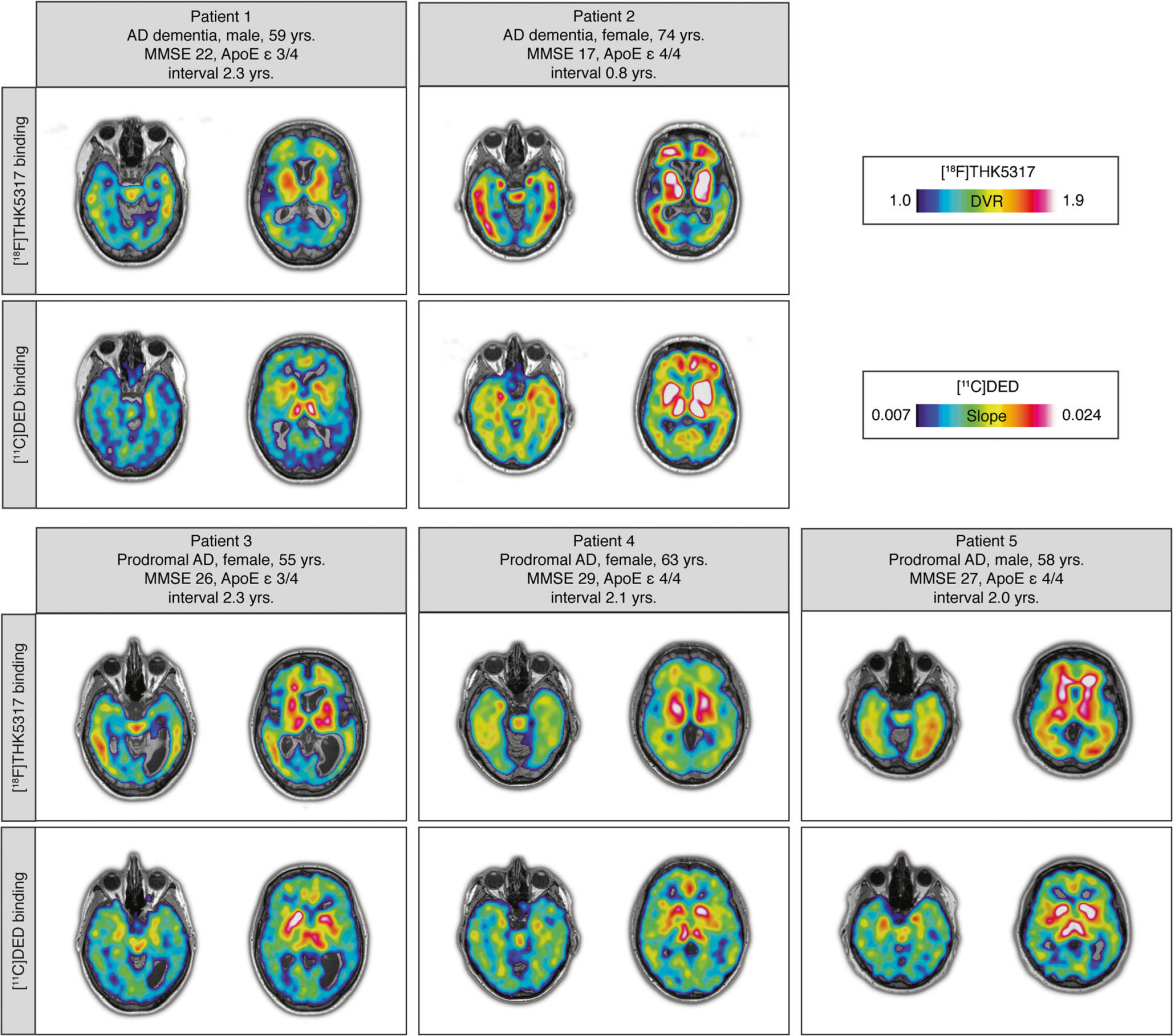
In vivo PET images with the tau tracer [^18^F]THK5317 and the MAO-B tracer [^11^C]DED in five patients with Alzheimer’s disease (AD; prodromal or dementia). The clinical characteristics of the patients are shown in the figure. ApoE = apolipoprotein; DVR = distribution volume ratio; interval = time interval in years between the PET scans with the tau tracer [^18^F]THK5317 and the MAO-B tracer [^11^C]DED; MMSE = mini-mental state examination

**Fig. 6:**
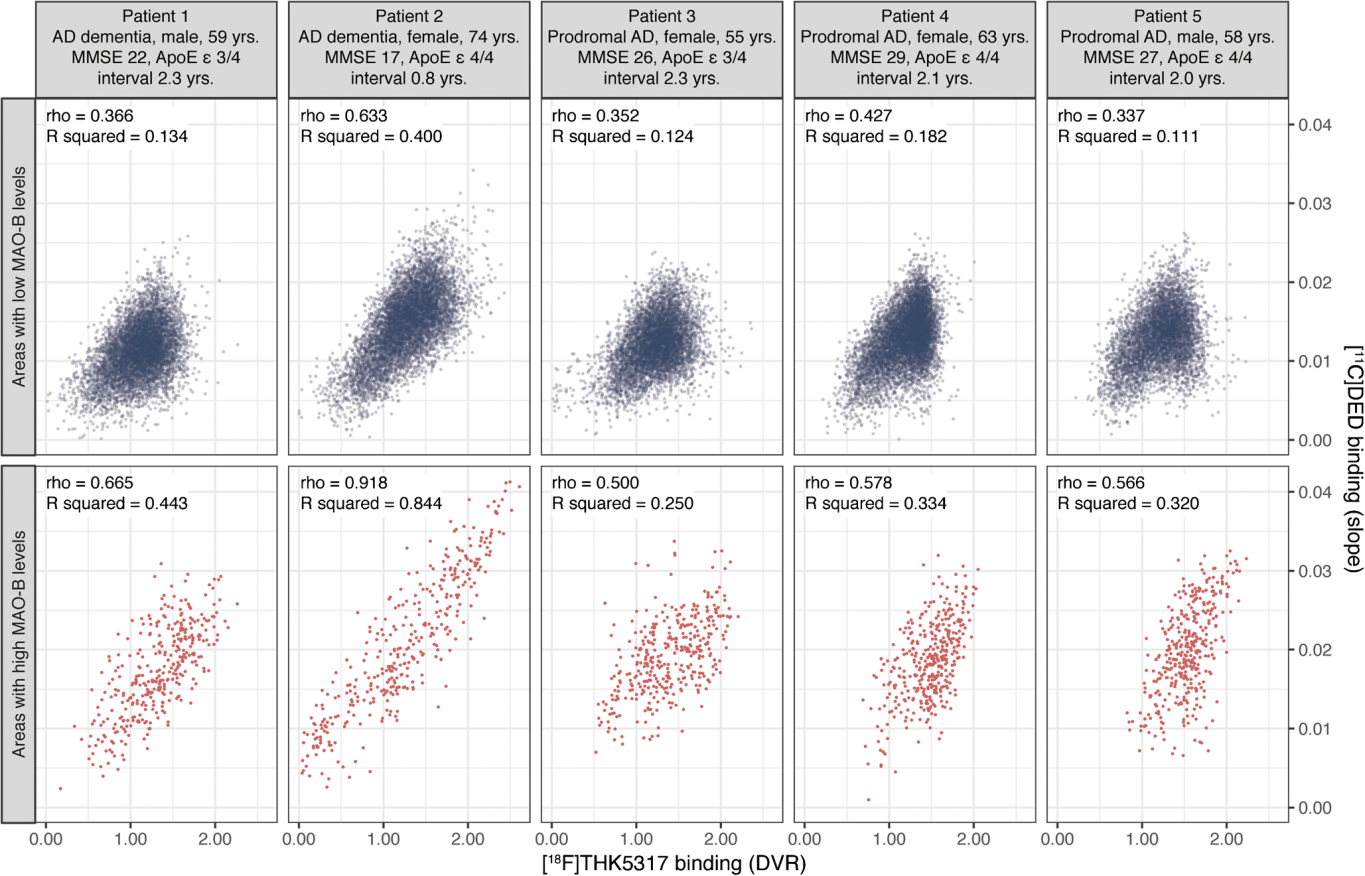
Within-patient voxel-wise Spearman correlations between in vivo tau [^18^F]THK5317 and MAO-B [^11^C]DED binding for each of the five patients with Alzheimer’s disease (AD; prodromal or dementia) when evaluated in brain areas with low MAO-B levels (*upper row*) and high MAO-B levels (*bottom row*). ApoE = apolipoprotein; DVR = distribution volume ratio; interval = time interval in years between the PET scans with the tau tracer [^18^F]THK5317 and the MAO-B tracer [^11^C]DED; MMSE = mini-mental state examination; rho = Spearman correlation coefficient; R squared = coefficient of determination

The clinical data for the included patients are shown in Figs. 5, 6 and Supplementary Table 3. For all patients, the most extensive cerebral binding for both [^11^C]DED and [^18^F]THK5317 was observed subcortically, in the basal ganglia and thalami. Of note, the additional binding of [^18^F]THK5317 in the midbrain and the appearing spillover of signal in the surrounding white matter results in discrete differences in the visual inspection of [^11^C]DED and [^18^F]THK5317 scans in the subcortical nuclei. The tracers showed binding in the isocortical temporal lobe and other isocortical areas and, although some agreement was observed between the tracers binding in individual brain areas, overall, the tracers had different regional binding distributions. More specifically, while [^11^C]DED binding was restricted mainly to the medial temporal lobe and the cingulate cortex, [^18^F]THK5317 binding extended to the lateral temporal, lateral frontal and parietal lobes (Fig. 5). Correlation analyses of the binding of the two tracers in individual patients showed weak-to-moderate relationships isocortically. Conversely, moderate-to-strong correlations were observed subcortically for all patients (Fig. 6). Although there was a consistent difference, in terms of correlation coefficients, between ROIs in all patients, the coefficients for the individual patients varied substantially.

The linear mixed-effects model highlighted the significant effect of [^11^C]DED binding on [^18^F]THK5317 binding across the whole GM [F (1, 71,025) = 12,412, *p* < 2.2e–16]. The interaction between [^11^C]DED binding and ROI was statistically significant [F (1, 71,026) = 180, *p* < 2.2e–16], indicating that there was a stronger relationship between [^18^F]THK5317 and [^11^C]DED binding in the subcortical ROIs, which have high MAO-B levels, than in the isocortical ROIs, which have low MAO-B levels. Moreover, a statistically significant interaction was observed between [^11^C]DED binding and the average GM [^11^C]DED binding per patient [F (1, 70,941) = 920, *p* < 2.2e–16], indicating that the strength of the relationship between [^18^F]THK5317 and [^11^C]DED binding depended on each individual’s [^11^C]DED binding load; a stronger relationship was observed between tracers with higher loads of [^11^C]DED binding. More details about the output of the linear mixed-effects model are available in Supplementary Table 1.

## Discussion

In this study, we employed computational modelling techniques for investigating the interaction of tau tracers with MAO-B, and we used PET imaging to evaluate the component of the in vivo tau tracer binding, which derives from this interaction. We found that all first-generation tau PET tracers showed similar binding affinity to MAO-B, comparable to that of a commonly used clinical MAO-B inhibitor. The in vivo regional binding pattern (distribution) of one of the first-generation tracers (i.e. [^18^F]THK5317) was, however, different overall from that of the studied MAO-B tracer ([^11^C]DED), although areas of suspected off-target binding to MAO-B were detected. The relationship between the two tracers with respect to binding depended largely on the availability of MAO-B enzyme in the different ROIs and on the varying brain MAO-B levels in patients with AD. The studied second-generation tau PET tracers (i.e. JNJ-311, MK-6240 and PI-2620) interacted less with MAO-B, possibly partly because of their low molar volume relative to the other tracers (Supplementary Table 2).

The substantial overlap of the structure of safinamide in the crystal and docked forms (see Fig. 1) suggests that the docking simulations were successful in locating the binding site in MAO-B, and that simulations like these can be used to predict the binding sites of other compounds. In the docking simulation, the safinamide benzylamino and propionamide groups extended over the substrate cavity site, and the fluorobenzyloxy group was located in the entrance cavity site [32]. The molecular docking studies illustrated that all tau PET tracers bind to the MAO-B enzyme with a binding affinity that is generally similar to that of the MAO-B inhibitor safinamide (inhibition constants in the nM range) and that safinamide and the tau tracers compete for the same binding site on the MAO-B enzyme. Furthermore, the binding affinities to MAO-B that were calculated were in close agreement with those calculated in vitro in ligand assays for safinamide [33] and the most widely used tau tracers (tracers of the THK family, T807) [3], which reinforces the translation of our computational modelling approach, at least to an in vitro situation. These results confirm the suspected MAO-B off-target binding of tau PET tracers and indicate that this is a common characteristic of all the developed tracers.

Nevertheless, even though molecular docking provides useful information about the number of binding sites and binding poses for the ligands in different binding sites of the biomolecular targets, the binding affinities predicted from this method are sometimes not that accurate, since docking uses single configuration of the protein or target and usually does not account for the ligand induced changes in the binding site. Therefore, it is often recommended to use molecular dynamics approaches with subsequent free energy calculation methods to investigate in a more precise manner the relative binding affinities of different ligands, which also incorporate measures of stability of the interactions between ligands and target. The discrepancy in the binding affinity measures from molecular docking (binding affinities and inhibition constants, Table 1) and molecular dynamics (free energies, Table 2) for the tracer MK-6240 towards MAO-B further illustrates the differences between the two techniques. MK-6240—a tracer for which preliminary in vitro and in vivo findings suggest low binding to MAO-B [15, 34]—shows affinity towards MAO-B comparable to the other tracers in the same binding site (molecular docking), but relatively low free energy of binding towards the same target (molecular dynamics and MM-GBSA), with the latter quantity serving as a measure of stability of the association process between tracer and the enzyme. These observations allow us to speculate that the tracer could interact with MAO-B, but would dissociate from the enzyme easier than the other first generation tau tracers (e.g. THK5317, THK5351, T807, PBB3), and would therefore have a lower overall binding to that off-target structure. Taken together, the molecular docking results should be interpreted with caution in light of the free energy calculations.

In more detail, it is apparent from the molecular dynamics based free energy calculation approach that the first generation tracers showed comparable relative binding affinity to MAO-B (as expressed in the free energies calculations) with the MAO-B inhibitor safinamide, while lower relative affinity was shown for the tracers THK523, RO-948 and T808. Even though we have not explicitly studied the GTP-1 tracer, its binding profile towards MAO-B should be similar to that of T808 since it has the same chemical structure as T808. The difference in its molecular weight, as it is dideuteriated, when compared to T808 will only affect the kinetics of binding but not the binding thermodynamics. Moreover, the most recently developed tau tracers (e.g. MK-6240, JNJ-311 and PI-2620) interacted the least with MAO-B of all the tracers (see the binding free energy values in Table 2), probably partly because of their relatively low molar volume, which does not favour their interaction with the binding site on the MAO-B enzyme (see the molar volumes of the investigated tau tracers in Supplementary Table 2). More specifically, the binding site of MAO-B is a tunnel-like microvolume [35] and ligands with a large molar volume can therefore interact with more residues around the tunnel-like cavity, maximising the magnitude of their binding free energy and hence their affinity. The relatively low cross interaction of the second generation tau tracers is in agreement with preliminary reports of the low binding of these tracers to the off-target basal ganglia [36, 37]; use of second generation tracers could offer substantial advantages in clinical tau PET with respect to potentially lower in vivo off-target binding.

The development of novel tracers is a rigorous and expensive process and using a molecular docking fast screening tool for investigating off-target binding to MAO-B, as discussed above, could be of great value. However, it is worth bearing in mind that the translation of computational modelling results to the in vivo situation is subject to a major limitation in terms of the in silico techniques. While binding affinities can be estimated in silico using simulations, the same does not apply to the tracer’s pharmacokinetic properties. Differences in these properties could play a fundamental role in any potential cross-interactions of a tracer with different targets, irrespective of the exact binding affinity. Therefore, since factors such as the tracer’s association/dissociation constants remain largely unexplored, it is difficult to assess the tracer’s off-target component based solely on the available simulation evidence, with the gap between in silico and in vivo remaining wide.

Since the in silico estimates provided evidence of a significant binding affinity between the tau tracers and MAO-B, we also explored the relationship between tau and MAO-B tracers using a complementary proof-of-concept study in five individuals who had both MAO-B [^11^C]DED and tau [^18^F]THK5317 PET scans. While the [^11^C]DED and [^18^F]THK5317 binding patterns were in agreement with the expected distribution of MAO-B and tau pathology, respectively [6, 26, 31, 38], [^18^F]THK5317 also showed extensive off-target binding to the basal ganglia and thalami, areas with high MAO-B and low tau loads, as has been observed previously in vivo and in vitro with various tau tracers [3, 11, 39]. Our findings indicate that the off-target component of the tau tracer, in this case [^18^F]THK5317 binding, is largely dependent on the concentration of the MAO-B enzyme in a given brain area. MAO-B could account for 11–18% (based on the calculated coefficients of determination) of the [^18^F]THK5317 binding in brain areas with low concentrations of the enzyme, and for much more (25–84%) in areas with higher MAO-B concentrations. Based on the regional distribution of MAO-B in the human brain, the areas with the highest concentrations (i.e. basal ganglia and thalami) do not overlap with the areas where tau pathology is primarily located in the AD brain but do overlap with those in non-AD tauopathies, such as corticobasal degeneration or progressive supranuclear palsy [38]. Therefore, although the existing tracers might not be optimal for differentiating between tauopathy syndromes in vivo, they might still be useful for following the progression of the pathology in AD. Interestingly, however, the load of MAO-B enzyme in the isocortex, as imaged with PET, appears to vary between and within individuals at different stages of AD, possibly as a result of reactive astrocytes in the human brain [27, 31], which adds to the complexity of in vivo imaging with the developed tracers, especially for the first-generation tracers.

It is interesting to compare the findings of our study with those of previous studies investigating the cross-interaction of tau tracers with MAO-B. Although recent in vitro studies agree on the existence of such a cross-interaction [3, 13, 14, 40], the results of the in vivo studies have been equivocal, probably because of the blocking design used, with the administration of irreversible MAO-B inhibitors [12, 41]. However, such a design is not optimal for this purpose, given the effects of MAO-B inhibitors on blood flow, and therefore the delivery of the tracers [42]. Our in vivo design, despite its inherent limitations as discussed below, represents an alternative to those approaches since it allows the assessment of the MAO-B component of the tracers in an unbiased manner.

The strength of this study lies in the investigation of the off-target binding of all the developed tau tracers to the MAO-B enzyme in a translational manner using initial computational modelling as well as an in vivo pilot analysis. However, it is important to bear in mind the possible bias of these approaches. Firstly, although computational analyses aim to accurately simulate the in vivo interactions between molecules and their targets, discrepancies between the computational and experimental results cannot be excluded because of the limitations of replicating the in vitro or in vivo conditions in silico. For example, although the computational analyses produced inhibition constants for the tau tracers and the reversible MAO-B inhibitor safinamide that were comparable to those of in vitro studies, our modelling approaches would not be able to simulate the binding of irreversible-suicide MAO-B inhibitors (i.e. selegiline, rasagiline) because the force-field approaches are unable to model association processes, which involve covalent bond formation. The currently used force-field method only captures the initial enzyme:ligand association process and it is after this event that the covalent bond is formed. Secondly, although studies directly comparing the in vivo binding of tau PET tracers with that of MAO-B tracers offer an optimal design for investigating the MAO-B component of tau tracers, the results of those studies need to be interpreted with caution because of their retrospective nature and the small sample sizes, which could bias the observations. Finally, the varying and often long intervals between [^18^F]THK5317 and [^11^C]DED investigations is another source of weakness in this study. Earlier studies, as mentioned above, have illustrated that [^11^C]DED binding declines with disease progression [27, 31] and therefore the decline in cognitive performance between investigations, although relatively mild in most patients of this sample (Supplementary Table 3), could limit the validity of our findings; had the [^18^F]THK5317 and [^11^C]DED investigations been performed at the same time point and with the same PET system, the strength of the association could have been somewhat different. Further work taking these observations into consideration is required to evaluate the clinical utility of the existing tau PET tracers, given their off-target binding, and to develop new tau tracers with improved pharmacokinetic properties.

## Electronic supplementary material

ESM 1 (DOCX 75 kb)
