# Supplementary material for: Cross-interaction of tau PET tracers with monoamine oxidase B: evidence from in silico modelling and in vivo imaging
Source: Eur J Nucl Med Mol Imaging. 2019 Mar 27;46(6):1369–82. doi: 10.1007/s00259-019-04305-8 (PMC6486902; doi:10.1007/s00259-019-04305-8)
Supplement: Supplementary file 1 — (DOCX 75 kb) [file 259_2019_4305_MOESM1_ESM.docx]

Supplementary Table 1. Analysis of variance for THK5317 binding with Satterthwaite's approximation to degrees of freedom (DF).

|  | **Sum Sq** | **Mean Sq** | **Num DF** | **Den DF** | **F value** | **p value** |
| --- | --- | --- | --- | --- | --- | --- |
| **Fixed factors** |  |  |  |  |  |  |
| DED | 755 | 755 | 1 | 71025 | 12412 | 2.2e-16* |
| ROI | 1 | 1 | 1 | 7 | 9 | 0.019* |
| Patients’ average DED | 1 | 1 | 1 | 7 | 15 | 0.006* |
| **Interaction terms** |  |  |  |  |  |  |
| DED:Patients’ average DED | 56 | 56 | 1 | 70941 | 920 | 2.2e-16* |
| DED:ROI | 11 | 11 | 1 | 71026 | 180 | 2.2e-16* |

DED = deuterium-L-deprenyl; Den = denominator; Num = numerator; ROI = region of interest; Sq = square.

Supplementary Table 2. Molar volumes of the monoamine oxidase-B inhibitor safinamide and the studied tau PET tracers.

|  | Molar volume (bohr^3^/mol) |
| --- | --- |
| **MAO-B inhibitor** | |
| Safinamide | 2511.017 |
|  |  |
| **Tau tracers** | |
| FDDNP | 2566.250 |
| PBB3 | 2544.728 |
| T807 | 2089.007 |
| T808 | 2303.010 |
| THK5105 | 2896.208 |
| THK523 | 2486.247 |
| THK5317 | 2705.628 |
| THK5351 | 2721.986 |
| RO-948 | 1881.733 |
| MK-6240 | 1903.865 |
| JNJ-311 | 1934.539 |
| PI-2620 | 1743.195 |

| **ID** | **Diagnosis** | | **MMSE** | | **PET system** | | **Treatment** | |
| --- | --- | --- | --- | --- | --- | --- | --- | --- |
|  | **DED** | **THK** | **DED** | **THK** | **DED** | **THK** | **DED** | **THK** |
| **Patient 1** | AD dementia | AD dementia | 22 | 17 | Discovery ST PET/CT (GE) | ECAT EXACT HR+ (Siemens) | Galantamine | Galantamine |
| **Patient 2** | AD dementia | AD dementia | 23 | | ECAT EXACT HR+ (Siemens) | ECAT EXACT HR+ (Siemens) | Galantamine | Galantamine |
| **Patient 3** | Prodromal AD | AD dementia | 26 | 23 | Discovery ST PET/CT (GE) | ECAT EXACT HR+ (Siemens) | Galantamine | Rivastigmine |
| **Patient 4** | Prodromal AD | Prodromal AD | 29 | 30 | Discovery ST PET/CT (GE) | ECAT EXACT HR+ (Siemens) | - | - |
| **Patient 5** | Prodromal AD | Prodromal AD | 27 | 28 | ECAT EXACT HR+ (Siemens) | ECAT EXACT HR+ (Siemens) | - | Donepezil |

Supplementary Table 3. Clinical research diagnosis, global cognitive performance (MMSE), and ongoing treatment for the five participants at the two time points of PET investigations (time points for [^11^C]DED and [^18^F]THK5317 PET). The PET system used at the two time points is also indicated.

For patient 3 only one neuropsychological assessment was performed due to the short interval between DED and THK PET investigations. DED = [^11^C]deuterium-L-deprenyl; MMSE = mini mental state examinations; THK = [^18^F]THK5317.
